# Correction to: Shp2 positively regulates cigarette smoke-induced epithelial mesenchymal transition by mediating MMP-9 production

**DOI:** 10.1186/s12931-022-02121-7

**Published:** 2022-08-24

**Authors:** Ya-nan Liu, Yan Guan, Jian Shen, Yong-liang Jia, Jian-cang Zhou, Yun Sun, Jun-xia Jiang, Hui-juan Shen, Qiang Shu, Qiang-min Xie, Yicheng Xie

**Affiliations:** 1 The Children’s Hospital, Zhejiang University School of Medicine, National Clinical Research Center for Child Health, Zhejiang, 310052 Hangzhou China; 2 Zhejiang Respiratory Drugs Research Laboratory of Food and Drug Administration of China, Zhejiang University School of Medicine, Zhejiang, 310058 Hangzhou China; 3 The First People’s Hospital of Yancheng, Yancheng, 224001 Jiangsu China; 4 Medical College of Yangzhou University, 11 Huaihai Road, Yangzhou, 225001 Jiangsu China; 5 Sir Run Run Shaw Hospital, Zhejiang University School of Medicine, Zhejiang, 310000 Hangzhou China; 6 Breath Smooth Biotech Hangzhou Co, LTD., Zhejiang, 310012 Hangzhou China

## Correction to: Respiratory Research (2020) 21:161 https://doi.org/10.1186/s12931-020-01426-9

Following publication of the original article [1], the authors identified an error in Fig. 6B. During the preparation of the figures in the above article, the authors regret that an error occurred during the assembly of Fig. 6B. Erroneous duplication of E-cadherin images were mistakenly assembled for MMP-9^+^ PHPS-1^+^ and MMP^+^ Shp2(siRNA)^+^ group.

The authors apologize for any inconvenience caused, and have confirmed that the conclusions were not affected.

The correct Fig. 6B is given in this correction article.

**Fig. 6:**
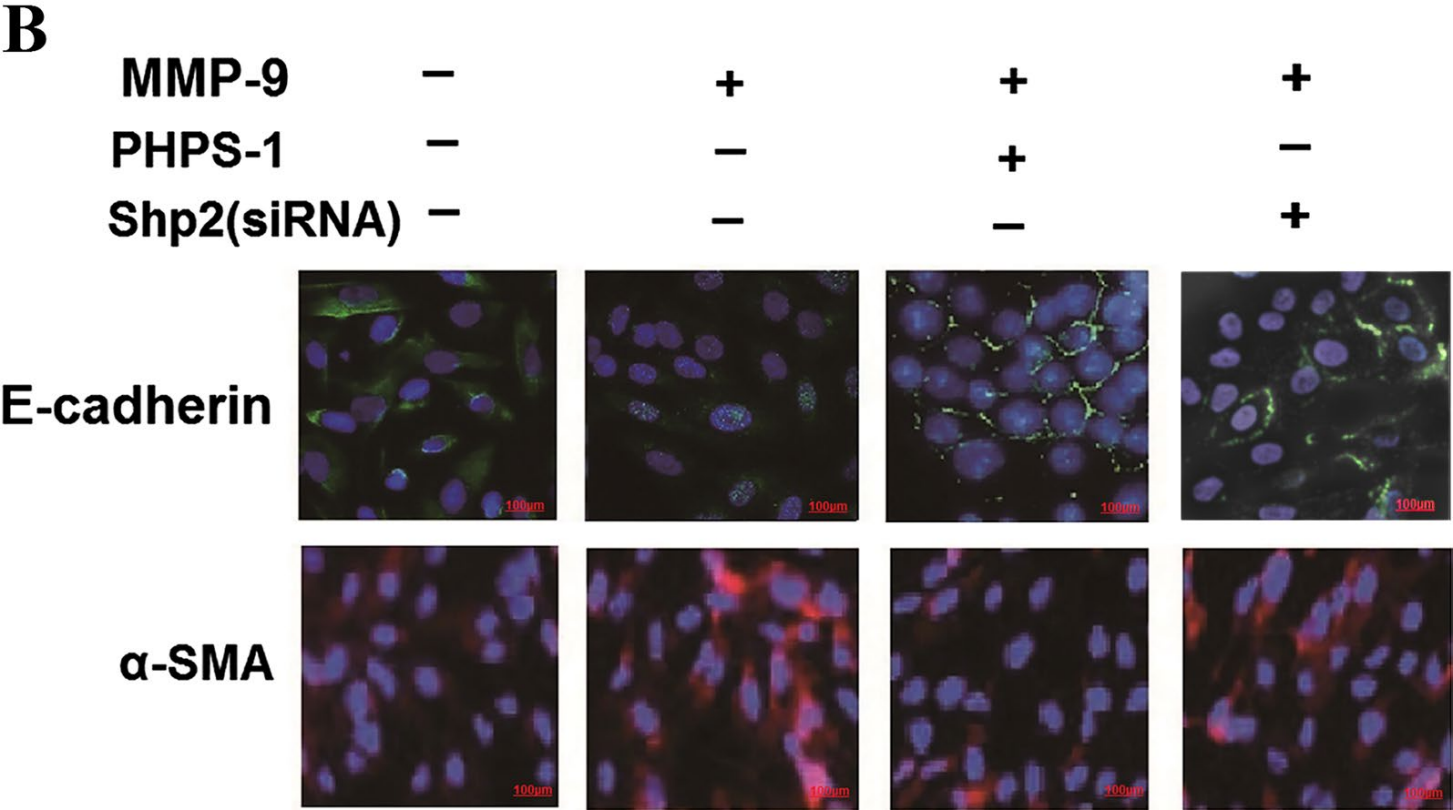
MMP-9 inhibition, Shp2 inhibition or Shp2 knockdown suppresses the expression of EMT-related factors induced by recombinant MMP-9 in NCI-H292 cells. **a** NCI-H292 cells with no treatment exhibit a pebble-like shape and display cell-cell contacts consistent with an epithelial morphology. The cells treated with human recombinant MMP-9 (2 μg/ml, 48 h) exhibit a fibroblast-like morphology with cellular elongation and reduction of cell-cell contacts. SB-3CT (1 μM) prevents the MMP-9-induced cellular changes and preserves normal epithelial morphology. The cells treated with recombinant MMP-9 for 48 h exhibit weaker expression of E-cadherin and stronger expression of α-SMA, compared with control. MMP-9 inhibition by SB-3CT (1 μM) alleviates the recombinant MMP-9 induced changes of E-cadherin and α-SMA expression. Scale bar = 100 μm. **b** Pharmacological inhibition or Shp2 knock down reverses the recombinant MMP-9 induced changes of E-cadherin and α-SMA expression. Scale bar = 100 μm. **c** MMP-9 inhibition by SB-3CT (1 μM) prevents the recombinant MMP-9 (2 μg/ml) induced decreases in E-cadherin expression and increases in α-SMA mRNA expression assessed by real-time PCR. *n* = 3 per group. ^#^*p* < 0.05 compared with control (no treatment); *p < 0.05 compared with cells treated with recombinant MMP-9. **d** Shp2 inhibition by PHPS1 (10 μM) or knock down by siRNA prevents the recombinant MMP-9 (2 μg/ml) induced decreases in E-cadherin expression and increases in α-SMA protein expression assessed by western blot. Data are expressed as mean ± SEM of three independent experiments. *n* = 3 per group. ^#^*p* < 0.05 compared with control (no treatment); *p < 0.05 compared with cells treated with recombinant MMP-9. At least three independent experiments were completed for each group assessment. Data are presented as the mean ± SEM. Statistical significance is determined by one-way ANOVA followed by the Student-Newman-Keuls test
